# Chinese patent medicine for functional dyspepsia effects: A protocol for systematic review and Bayesian network meta-analysis

**DOI:** 10.1097/MD.0000000000027761

**Published:** 2021-11-24

**Authors:** Jingjie Wu, Yao Wei, Yaoxin Chen, Yu Long, Nierui Huang, Yingbing Mei

**Affiliations:** a Shanghai Jinshan TCM-integrated Hospital, Shanghai, China; b Hubei Provincial Hospital of TCM, Wuhan, China; c Shanghai University of Traditional Chinese Medicine, Shanghai, China; d Huanggang Central Hospital, Huanggang, China.

**Keywords:** Chinese patent medicine, functional dyspepsia, network meta-analysis, traditional Chinese medicine

## Abstract

**Background:**

In recent years, many clinical studies have suggested that various Chinese patent medicines have the potential to treat functional dyspepsia (FD). This study aims to conduct a systematic review and Bayesian network meta-analysis to evaluate the effectiveness of different Chinese patent medicines for FD.

**Methods:**

A comprehensive retrieval method will be executed in the following databases: PubMed, Web of Science, Embase, Cochrane Library, China National Knowledge Infrastructure (CNKI), China Biology Medicine disc (CBM), VIP Database, and Wanfang Database. Clinical randomized controlled trials (RCTs) of 9 Chinese patent medicines for FD are searched, and the retrieval time is from inception to October 2021. Three reviewers will screen the RCTs that meet the inclusion criteria and extract the data independently. The outcomes include total clinical efficiency, cure rate, recurrence rate, symptom score, and adverse events. Cochrane risk-of-bias tool will be carried to assess RCTs quality. The “gemtc” package and “rjags” package in R software will be used to manage data within the Bayesian framework.

**Results:**

The results can provide relatively objective evidence to evaluate the effectiveness of these 9 Chinese patent medicines in treating FD, which may help clinicians to develop a more effective and safer treatment plan.

**Conclusion:**

This study aims to provide new options for Chinese patent medicine treatment of FD in terms of its efficacy and safety.

## 1 Introduction

Functional dyspepsia (FD) is one of the most common diseases in gastroenterology outpatient, and its prevalence can be as high as 10% to 30%, which is a worldwide public health problem.^[1]^ According to the Rome IV criteria, FD is diagnosed with symptoms centered in the gastroduodenal area, including epigastric pain, epigastric burning, postprandial fullness, and early satiation, in the absence of any organic, metabolic, or systemic disease that can explain the symptoms.^[2]^ FD is not life-threatening, but it has a great impact on the quality of daily life and is easy to recur, which may cause huge psychological and physical damage to patients. The underlying pathology of FD is not fully understood, it is considered to be related to upper gastrointestinal inflammation, gastrointestinal motility and sensory dysfunction, visceral hypersensitivity, Helicobacter pylori infection, gastrointestinal hormones, brain-gut axis, etc.^[3,4]^ Conventional approaches to FD include prokinetic drugs, Helicobacter pylori eradication, proton pump inhibitors, and antidepressant therapy.^[2,5]^ However, due to the unclear pathology, single pharmacotherapy in treating FD usually cannot manage the symptoms fully, and long-term treatment may lead to serious effects.^[6,7]^

Nowadays, interest in complementary and alternative medicine, such as traditional Chinese medicine (TCM), is increasing. Under the guidance of TCM theory, FD is caused by spleen deficiency, qi stagnation, and stomach disharmony. Therefore, invigorating spleen, stomach, and qi can strengthen the body's resistance to manage symptoms. Chinese patent medicine is a type of TCM product that is approved by the China Food and Drug Administration and appeared on the market. An increasing number of studies have shown the effectiveness of various Chinese patent medicines in the treatment of FD,^[8–10]^ but there is no comparison between these Chinese patent medicines. In this study, we aim to evaluate the efficacy of 9 Chinese patent medicines for FD and provide explicit guidance for clinicians.

We present the following article in accordance with the Preferred Reporting Items for Systematic Review and Meta-Analysis Protocols (PRISMA-P) reporting checklist.

## 2 Methods

### 2.1 Eligible criteria

#### 2.1.1 Types of studies

Randomized controlled trials of Chinese patent medicine for FD will be included, whether blind or not. Languages are limited to Chinese and English. Reviews, case reports, conference papers, dissertations, animal studies, basic research, studies unrelated to Chinese patent medicine or FD will be excluded.

#### 2.1.2 Types of participants

For patients diagnosed with FD, the diagnostic criteria include Rome II, Rome III, and Rome IV. The sample characteristics (age, gender, course of disease, etc) between the control group and the treatment group should be consistent.

#### 2.1.3 Types of interventions and comparisons

The treatment group is treated with 9 kinds of Chinese patent medicines included: Zhizhu Kuanzhong capsule, Qi-zhi-wei-tong granule, Wei-chang-an pill, Biling Weitong Granules, Dalitong granule, Si-mo-tang oral liquid, Liuwei Nengxiao capsule, Liuwei Anxiao capsule, Huoxiang Zhengqi capsule/pill. The control group is treated with placebo or western medicine (domperidone, trimebutine, itobili, moxabili, and omeprazole). The dosage and course of treatment are unlimited.

#### 2.1.4 Types of outcome measures

The main outcomes will be total clinical efficiency, cure rate, recurrence rate, symptom score, and adverse events. If related data are sufficient, gastric motility indicators and serum gastrointestinal hormones, such as gastric emptying rate and motilin, will be further analyzed.

### 2.2 Search strategy

Comprehensive retrieval will be carried out in the PubMed, Embase, Cochrane Library, Web of Science, China National Knowledge Infrastructure (CNKI), Chinese Biomedical Database, VIP Database, and Wanfang Database, from inception to October 2021. Search terms include functional dyspepsia, the names of different Chinese patent medicines, and their synonyms. The search strategy for each database will be properly modified. The detailed search strategy for PubMed is presented in Table 1.

**Table 1:** Search strategy for PubMed.

#1	functional dyspepsia [MeSH Terms]
#2	nonulcer dyspepsia [Title/Abstract]
#3	functional gastrointestinal disorder [Title/Abstract]
#4	postprandial distress syndrome [Title/Abstract]
#5	epigastric pain syndrome [Title/Abstract]
#6	functional dyspepsia [Title/Abstract]
#7	#1 OR #2 OR #3 OR #4 OR #5 OR #6
#8	Chinese patent medicine [Title/Abstract]
#9	zhizhukuanzhong [Title/Abstract] OR zhizhu kuanzhong [Title/Abstract] OR zhi-zhu-kuan-zhong [Title/Abstract]
#10	qizhiweitong [Title/Abstract] OR qizhi weitong [Title/Abstract] OR qi-zhi-wei-tong [Title/Abstract]
#11	weichangan [Title/Abstract] OR wei-chang-an [Title/Abstract]
#12	bilingweitong [Title/Abstract] OR bi-ling-wei-tong [Title/Abstract] OR biling weitong [Title/Abstract]
#13	simotang [Title/Abstract] OR si-mo-tang [Title/Abstract]
#14	dalitong [Title/Abstract] OR da-li-tong [Title/Abstract]
#15	liuweinengxiao [Title/Abstract] OR liuwei nengxiao [Title/Abstract] OR liu-wei-neng-xiao [Title/Abstract]
#16	liuweianxiao [Title/Abstract] OR liuwei anxiao [Title/Abstract] OR liu-wei-an-xiao [Title/Abstract]
#17	huoxiangzhengqi [Title/Abstract] OR huoxiang zhengqi [Title/Abstract] OR huo-xiang-zheng-qi [Title/Abstract]
#18	#8 OR #9 OR #10 OR #11 OR #12 OR #13 OR#14 OR #15 OR #16 OR #17
#19	#7 AND #18

### 2.3 Data extraction

**Figure 1:**
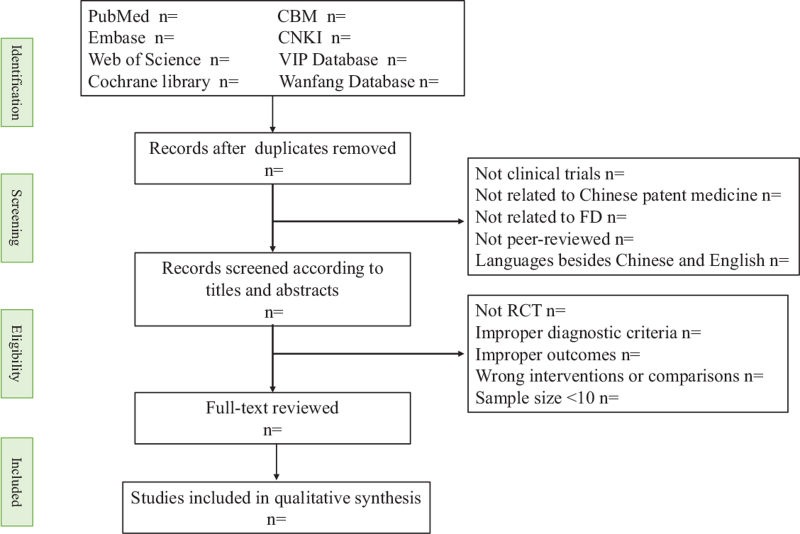
PRISMA flow diagram of selection process.

Screening and data extraction were carried out independently by 2 reviewers. If there is any controversy, it would be resolved by a third reviewer. A PRISMA flow diagram will be established to describe the whole selection process (Fig. 1).

Two reviewers will independently extract the study characteristics and outcome data using the same data extraction criterion. The following information will be included: basic information of randomized controlled trials: title, year of publication, first author, country; participants: gender, age, sample size; intervention: names of the intervention in control group and treatment group, treatment duration; details of trial design: randomization, blinding, allocation concealment, and so on; outcome measures: total clinical efficiency, cure rate, recurrence rate, symptom score, and adverse events.

### 2.4 Risk of bias assessment

Two reviewers will use the Cochrane Risk of Bias Tool to evaluate the quality of all filtered studies, and the third reviewer will settle disputes. There are 6 sources of bias including randomization process, deviations from intended interventions, missing outcome data, measurement of the outcome, selection of the reported result, and other bias. The risk of bias can be divided into 3 degrees: low risk, high risk, and some concerns.

### 2.5 Statistical analysis

#### 2.5.1 Pairwise meta-analysis

If there are no fewer than 3 studies of the same interventions and outcome measures, the pairwise meta-analysis will be performed with Review Manager software version 5.3 (The Nordic Cochrane Center, The Cochrane Collaboration, 2014, Copenhagen, Denmark) to provide direct evidence. For dichotomous variables, odds ratio and 95% confidence intervals will be adopted, while for continuous variables, outcomes will be expressed as standard mean difference and 95% confidence interval. I^2^ test will be used to assess heterogeneity. In the case of I^2^ < 50% and *P* > .05, the fixed-effects model will be used. Conversely, a random-effects model will be adopted.

#### 2.5.2 Network meta-analysis

In this study, the “gemtc” package and “rjags” package supported by R software version 4.0.4 (R Development Core Team) will be performed to present the results of network meta-analysis. The Markov chain Monte Carlo method will be used to combine both direct and indirect evidence for a particular endpoint. Four Markov chains will be used to set the model with an initial value of 2.5. We will set 20,000 simulations for each chain as the “burn-in” period, then yielding 50,000 iterations to obtain the odds ratios. When the potential scale reduction factors tend to 1, the model convergence is satisfactory; otherwise, the number of iterations will be increased. We will also calculate the rank probabilities, which can show the hierarchical position of each treatment. Brooks-Gelman-Rubin diagnosis plots, density plots, and trace plots were adopted to assess model convergence.

### 2.6 Publication bias

Funnel plots produced by RevMan software will be selected to assess the publication bias, which can display the relationship between effect size and sample size. In funnel plots, trials data distributed asymmetrically around the mean effect size show that there is publication bias.

### 2.7 Quality assessment

We use the Grades of Recommendations Assessment Development and Evaluation guideline to evaluate the quality of evidence. The guideline divides the quality of evidence into 4 degrees: high, moderate, low, and very low.

### 2.8 Ethics and informed consent

This protocol has been registered on INPLASY (https://inplasy.com/) with the registration ID INPLASY2021100057. And because this study is a protocol for systematic review, ethical approval can be skipped.

## 3 Discussion

FD is a common functional gastrointestinal disorder. According to different symptoms, FD can be divided into epigastric pain syndrome and postprandial distress syndrome.^[11]^ A systematic review of the global epidemiology of FD showed that the prevalence rate of FD was 21%, and its risk factors included female, smoking history, Hp infection, and non-steroidal anti-inflammatory drugs.^[12]^ The high incidence and recurrence rate of FD seriously disturb the daily life of patients.

Herbal medicine, as an important complementary and alternative medicine, has been proven to own certain advantages in treating FD.^[13,14]^ Chinese patent medicine as a type of TCM product uses herbal medicine as raw material and is processed into certain dosage forms according to the prescribed prescription. It is a commercialized preparation approved by the China Food and Drug Administration.^[15,16]^ Since an increasing number of Chinese patent medicines in treating FD appeared, making clinical application a difficult decision as it remains inconclusive which Chinese patent medicine can obtain the greatest benefits. Therefore, we decide to rank the efficacy of these 9 Chinese patent medicines by network meta-analysis. The results of this protocol will be published in the relevant journal as soon as possible, and a quick update will be made when supplements are required.

## Author contributions

**Conceptualization:** Jingjie Wu.

**Data curation:** Jingjie Wu, Yao Wei.

**Formal analysis:** Jingjie Wu.

**Investigation:** Jingjie Wu, Yu Long, Nierui Huang.

**Methodology:** Jingjie Wu, Yu Long.

**Project administration:** Jingjie Wu, Yu Long, Nierui Huang, Yingbing Mei.

**Resources:** Jingjie Wu, Yingbing Mei.

**Software:** Jingjie Wu, Yu Long.

**Supervision:** Yao Wei, Yaoxin Chen.

**Validation:** Yao Wei, Yaoxin Chen.

**Visualization:** Jingjie Wu.

**Writing – original draft:** Jingjie Wu, Nierui Huang.

**Writing – review & editing:** Yaoxin Chen, Yingbing Mei.
